# Discretion, power and the reproduction of inequality in health policy implementation: Practices, discursive styles and classifications of Brazil's community health workers

**DOI:** 10.1016/j.socscimed.2019.112551

**Published:** 2019-12

**Authors:** João Nunes, Gabriela Lotta

**Affiliations:** a Department of Politics, University of York, Heslington, York, YO10 5DD, UK; b Department of Public Management, Getúlio Vargas Foundation, Av Nove de Julho, 2029, São Paulo, Brazil

**Keywords:** Brazil, Community health workers, Power, Policy implementation, Health inequality, Street-level bureaucracy, Agentes comunitários de saúde

## Abstract

This article explores the mobilization of power by health workers during policy implementation, showing how in a context of discretion and resource scarcity they can reproduce inequalities in access to health services. The argument innovates theoretically by supplementing the ‘street-level bureaucracy’ literature, which emphasizes frontline worker discretion, with a conceptualization of power as domination encompassing the shaping of behavior, the constitution of subjects and the reproduction of inequality. Empirically, the article focuses on Brazilian community health workers (*agentes comunitários desaúde*, CHWs). CHWs are a neglected but highly important segment of the health workforce that traditionally functions as a link between the health system and disadvantaged groups. The article examines how Brazilian CHWs act as street-level bureaucrats mobilizing power in their interactions with users. They operate within a severely under-resourced public health system, the *Sistema Único de Saúde*, which places constraints upon their action and forces them to make allocation decisions with little training and support. The article highlights the ways in which inequalities in access to health services are reproduced (inadvertently or not) through the practices, discursive styles and classifications of CHWs. Methodologically, the paper is based on ethnography with 24 CHWs and interviews with 77 other CHWs in Brazil.

## Highlights

• Develops a novel framework for analysing health worker discretion and power.
• Supplements the street-level bureaucracy approach with the concept of domination.
• Explores the mobilization of power by Brazilian community health workers.
• Shows how policy implementation can reproduce inequality in access to healthcare.

## 1 Introduction

Notwithstanding the constraints they encounter, health workers are in many respects powerful. They hold knowledge and resources that impact directly on the lives and wellbeing of populations (Feather and Johnstone, 2001; Møller, 2009). Their insider position in the health system puts them in a privileged position to influence behavior (Skinner et al., 2007). Their ability to determine who can access information and health system resources means that they can contribute to redressing or reproducing inequalities. Power is thus an important dimension in the practice of health workers. The way they use power shapes the implementation of health policies and is crucial for their success or failure.

Sociological and public health scholarship has considered how physicians, nurses and patients are involved in power relations (Nettleton, 1992; Hewison, 1995; Henderson, 2003). Historical studies have shown how physicians and other health practitioners became powerful in colonial endeavors (Watts, 1997; Anderson, 2006). Nonetheless, power remains neglected in health systems analysis and policy discussions (Erasmus and Gilson, 2008; Sriram et al., 2018). The recent *Global Strategy on Human Resources for Health* provides only vague assertions about the need to ensure that health workers are ‘motivated and empowered to deliver quality care’ (World Health Organization, 2016, 10). In this document, health workers are overwhelmingly considered apolitical actors involved in neutral and value-free relations with patients. Power has remained largely undertheorized and understudied in what pertains to health worker practice in contemporary settings. It has not been systematically considered when monitoring health policy implementation and assessing obstacles therein.

The neglect of health worker power epitomizes a broader underestimation of the practice of policy implementation (Sheikh et al., 2014; Van Belle et al., 2017). This underestimation obscures the broader relations in society that shape, and are in turn shaped by, the implementation of health policies. It also blinds us to the complex process of implementation, which in some cases reveals substantial deviations from the original design of policies. Some studies have shown that the mobilization of power by health workers produces side-effects and unintended outcomes (Lehmann and Gilson, 2013, Harris et al., 2014; Scott et al., 2017). Reengaging power in health worker practice would help to address one of the most important puzzles in policy analysis: the gap between the original design, as conceived by formulators, and the materialization of policies by a chain of implementers (Pressman and Wildavsky, 1973; Hill and Hupe, 2003).

This article speaks to the relative neglect of health worker power, and hence to existing blind-spots regarding the practice of health policy implementation. We address the following questions: how can the power of health workers be conceptualized? How do health workers mobilize power in day-to-day practice, and with what consequences? Specifically, to what extent does power contribute to differentiated treatment and unequal access to health services and resources? Access is an important question given the pivotal role given to health workers in the drive to universal healthcare (World Health Organization, 2008) and the Sustainable Development Goals (de Francisco Shapovalova, 2015). The extent to which health workers contribute to addressing or reproducing inequalities in access to services is of crucial importance for the future of the global health agenda.

To answer these questions, we develop a new framework for the analysis of health worker power. We take as our starting-point the ‘street-level bureaucracy’ approach, which scrutinizes the day-to-day practices and interactions of implementers. As proponents of this approach note, implementation depends on routine judgments, decisions and actions of frontline workers, who have significant discretion to adjust policies to the local context in the face of emerging constraints and demands. Nonetheless, this approach does not sufficiently consider the role of power in street-level interactions. Aiming for a richer account of health worker practice, we supplement street-level bureaucracy with a multilayered notion of power as domination. The concept of domination enables us, first, to recognize the impact of health worker judgements and actions in shaping the behavior of health system users. Domination also encompasses the deeper effects of health worker practice in helping to constitute desirable subjects, in line with recommendations issued by the health system. Finally, domination allows us to conceive implementation as accompanied by the reproduction of inequalities. Bringing together street-level bureaucracy and domination, we provide a nuanced engagement with health worker power and its (often unintended) effects in terms of access to health services.

Empirically, we focus on the power of community health workers (CHWs), a segment of the workforce largely invisible in health policy discussions. This neglect is at odds with their importance. CHWs have been routinely presented as essential to healthcare delivery and to the redressing of inequalities in access to health services, particularly in low- and middle-income countries. CHW is the name given to close-to-community providers with no specialized medical training who traditionally operate as links between doctors, nurses and the population (Witmer et al., 1995; Olaniran et al., 2017). They can specialize in one task or carry out a diversity of functions, such as identifying health needs, particularly of neglected groups; gathering epidemiological data; scheduling consultations; supporting patients in long-term treatment and rehabilitation; supporting vaccination programmes and vector-control; and health education and disease prevention. Speaking to a burgeoning literature on the lives and experiences of CHWs (Oliver et al., 2015; Mlotshwa et al., 2015; Maes, 2017), we explore the role of CHWs in health policy implementation by looking at their practices (what they do in their day-do-day work); their discursive styles (how they interact with health system users using particular words and expressions); and their classifications (how they make decisions based on judgements about the behavior of users). We highlight the problematic aspects of CHW activity by showing how it is traversed by power. Domination is visible in CHW efforts to shape the behavior of users and constitute them as ‘healthy subjects’. Domination is also present in the reproduction of inequalities in access to health services, which emerge (inadvertently or not) through the practices, discourses and classifications of CHWs.

Brazil is an ideal setting for exploring how CHWs mobilize power in the context of a public health system. The country faces great demands for public policy because of profound inequalities in access to affordable healthcare. Brazil has a complex epidemiological profile combining so-called ‘diseases of poverty’ (such as mosquito-borne diseases or parasitic infections) with diseases prevalent in more developed nations (such as chronic diseases, obesity and heart disease). Brazil's health system rests upon an uneasy balance between the goal of universality and equity in access, and the need for policies that consider heterogeneity along territorial, socioeconomic, cultural, ethnic and racial lines. Brazilian CHWs have a central role in the government's drive to broaden access to health services, but they occupy an ambiguous position. On the one hand, they are given momentous tasks related to delivering frontline healthcare to vulnerable and remote populations, while seeking to alter their behaviors and promote visions of what a healthy citizen should be. In a context of widespread poverty and vulnerability, they emerge as powerful actors because of their access to resources and their ability to influence community-level health outcomes. On the other hand, CHWs work within a severely under-resourced health system, which forces them to make resource allocation decisions without adequate training and based on their own judgements about the merits of health system users. The context of resource scarcity and CHW discretion leads to the mobilization of power in ways that reproduce inequalities in access to health services.

## 2 ‘Street-level bureaucracy’, power and domination in health policy analysis

The practice of policy implementation is a core concern of the ‘street-level bureaucracy’ (SLB) approach (Lipsky, 2010 [1980], Hupe et al., 2015). This approach focuses on street-level bureaucrats – such as police officers, teachers or health workers – who work in direct contact with citizens and mediate their day-to-day relations with authorities. More than mere conduits, bureaucrats have considerable autonomy and decision-making powers. These stem from the tension between rules and the realities on the ground, which often require negotiation and the exercise of judgment (Møller, 2016). Bureaucrats encounter ambiguities and uncertainties, and frequently need to exercise discretion to determine how the policy is to be implemented (Hupe and Hill, 2007). Policies are therefore framed, and can be altered, by the discretion of implementers.

The notion of discretion denotes the leeway given to bureaucrats to adapt rules to circumstances, in ways that are not always consistent with directives and goals (Maynard-Moody et al., 2003). It is limited since it does not capture the strategic logic inherent to the day-to-day activity of these workers, and the ways in which they exercise power to shape the meaning of policies and produce effects beyond what was originally envisaged. Overwhelmingly, the SLB literature sidesteps a thorough exploration of power by assuming that it stems naturally from the fact of discretion. It ends up providing a restrictive view of the role of implementers as power brokers, overlooking different modalities and effects of power present in discretionary decisions and actions.

The SLB approach has been applied to health systems research (Erasmus, 2014). Examples include studies of Ghanaian community-based providers (Atinga et al., 2018) and environmental health officials (Crook and Ayee, 2006); frontline nurses in Denmark (Harrits and Møller, 2014) and South Africa (Walker and Gilson, 2004); general practitioners in the United Kingdom (Checkland, 2004); and family planning services in Kenya (Kaler and Watkins, 2001). These studies consider health workers' experiences and perceptions in the face of challenges such as encroaching privatization or changing patient expectations. Here, too, there are limits to the SLB over-reliance on discretion. Noting the limited engagement with the discretionary power of front-line health professionals, Gilson et al. (2014, iii52) have called for analyses of the ‘micropractices of power’ influencing implementation.

The SLB focus on practice needs to be supplemented by a consideration of the power permeating street-level work. The notion of domination offers a useful starting-point, since it encompasses three modalities of power with different effects (Nunes, 2014). The first modality is the shaping of behaviour, not merely by coercion or threat but also by attempts to define what is appropriate or acceptable, and by foreclosing alternatives or dissent (Lukes, 2005). In the second modality, power emerges as a productive force ‘shaping and governing the capacities, competences and wills of subjects’ (Rose, 1996, 58). Power is not simply about prohibiting, but rather constituting subjects in line with visions of what a ‘good’ or ‘productive’ society should look like. Instead of conceiving individuals as mere targets of power, this view sees power as crucial for turning individuals into subjects of a well-ordered society (Foucault, 2008 [2004]). These two modalities need to be embedded in a structural context. Power relations occur in, and are themselves involved in the reproduction of, an uneven field in which certain groups are systematically placed in a position of subordination in relation to others (Young, 2011 [1990], Lovett, 2010). A third modality of power can thus be identified when considering the structural conditions that allow for certain behaviours and subjects to be promoted while others are prohibited or constrained, resulting in hierarchical and unequal outcomes.

Domination supplements discretion in the SLB approach by enabling an examination of how power operates in the daily practice of bureaucrats: through attempts to shape the behaviour of policy beneficiaries; through injunctions to constitute subjects who are predisposed to think and act in certain ways; and by reinforcing unequal relations in society. This has important implications for health policy analysis, since it reveals frontline health workers as nodes in the circulation of power relations that go beyond coercion, threat or punishment. The activity of health professionals can be approached against the background of attempts to influence behaviour and constitute ‘healthy’ subjects in line with visions of politically and economically useful citizenship (Nadesan, 2010).

With this framework, health worker practice can be embedded in context, which is often one of ambiguity. On the one hand, health professionals are powerful due (at least partly) to their position in the health system. This gives them access to expert knowledge that is used to improve health or prevent disease, but also mobilized in ‘gatekeeping’ roles when making resource allocation decisions (Feather and Johnstone, 2001). On the other hand, health workers face structural constraints. Foremost among these is the health system itself, which often grapples with resource insufficiencies, coordination problems and policy design deficiencies. Moreover, health workers are impacted by the broad political and socioeconomic setting, which determines the resources available and how they are distributed. By focusing on the practice and power of health workers, this framework takes their agency seriously, revealing nuance and complexity in daily interactions. Importantly, agency is shaped and limited by structural constraints, although never fully determined.

Another added value for health policy analysis of combining the SLB approach with the notion of domination is that the former enables discussions of power to be firmly grounded on day-to-day practice, thus avoiding generalizations about how power works and what its effects are. Thus, our framework does not assume the existence of a consciously dominating group. The reproduction of inequality can be an unintended consequence, a side-effect or the result of unconscious bias (Hearn, 2008). We do not assume that health professionals are necessarily motivated by a malign intention to control, acquire advantage for themselves or disadvantage others. Instead, our ‘bottom-up’ approach begins from what health workers do and how they interact with the beneficiaries of policies, against the background of constraints imposed by the health system and the socioeconomic context.

In sum, combining the SLB approach with a notion of power as domination provides a structured yet non-deterministic lens with which to explore the diverse modalities and impacts of health worker practice. The remainder of this article applies this framework to the case of Brazilian community health workers (CHWs). It examines the context of health policy implementation in Brazil; applies a bottom-up perspective to the activity of CHWs; and shows how this activity is traversed by power.

## 3 Community health workers in Brazil: practices, discourses and classifications

### 3.1 Context of health policy implementation in Brazil

Brazilian community health workers (*agentes comunitários de saúde*) are part of the Family Health Strategy (*Estratégia Saúde da Família*, ESF), a primary care programme within the (public) Unified Health System (*Sistema Único de Saúde*, SUS). The ESF aims to humanize healthcare, reduce inequalities and address risk factors, supplementing curative medicine in hospitals with close-to-community prevention, health promotion and rehabilitation. It is responsible for referral to other levels of care and thus functions as a gatekeeper in the SUS.

Brazil’s 1988 Constitution lay the general principles for the SUS: universal access, the reduction of inequalities and the safeguarding of diversity. As Brazil is a federal state, policies are normally designed and financed by the federal government, with municipalities being responsible for co-funding, adaptation and implementation. Given the great territorial, socioeconomic, cultural and ethnic heterogeneity of the country, federal guidelines are often generic. Even though there is general agreement on the nature and goals of policies, Brazil witnesses high levels of ambiguity in implementation, with great scope for health worker discretion and power. This, combined with chronic under-funding, coordination problems and demands not originally envisaged, has generated what Richard E. Matland (1995, 166) termed ‘experimental implementation’, in which ‘outcomes depend heavily on the resources and actors present in the microimplementing environment’. The result is significant local-level variation.

ESF interventions are undertaken by teams comprising general practitioners, nurses, nursing assistants and CHWs. Each team is responsible for an area encompassing 600 to 800 families (Cordoba and Jeronimo, 2013). There are around 20.000 teams in 80% of Brazilian municipalities, reaching out to 65 million citizens. To ensure that services are provided directly to the families, the program relies heavily on the frontline work of CHWs recruited from the communities. Each CHW follows a roughly equal number of families, regardless of the population size of their territory. The role of CHWs is to bridge the health system and its users, particularly marginalized and vulnerable groups. Their responsibilities include: health education and promotion; keeping records of individuals and families, identifying those at risk; making regular household visits to monitor the vaccination of children or the welfare of chronic patients; scheduling maternal health specialist appointments; advising on the correct use of medication; and contributing to mosquito-control campaigns (Ministério da Saúde, 2012).

There are currently over 264.000 CHWs in Brazil (Ministério da Saúde, 2018). CHWs can be hired through public procurement or outsourced, with different selection processes and contractual arrangements – permanent, temporary, verbal and informal contracts, bursaries, among others (Lima and Cockell, 2008). As CHWs come from the communities where they work, often they are providers and users in the same healthcare facilities. Proximity with the community means that they experience first-hand the health and socioeconomic vulnerabilities they are tasked with addressing. They are overwhelmingly women, with percentages above 75% and in some cases up to 95% (Musse et al., 2015; Simas and Pinto, 2017). Although the law requires CHWs to have at least nine years of formal education, the same studies reveal that 65% have completed secondary-level education. Nonetheless, CHWs are the less professionalized bureaucrats in the ESF. This is compounded by insufficiencies in training, which is fragmented, uneven across the country, reliant on short courses focused on specific interventions and often deployed when CHWs are already on the job or in response to ongoing crises (Morosini, 2010; Fonseca, 2016). This means that CHWs are often unprepared to exercise their discretion when responding to multiple demands and making decisions on how best to use the system's resources.

### 3.2 Data collection and analysis

A two-pronged research strategy was applied to investigate how Brazilian CHWs exercise power. Between 2008 and 2010 an ethnographic study was conducted with 24 CHWs in three municipalities: Sobral (population 140.000), Taboão da Serra (240.000) and São Paulo (11 million). Eight CHWs were selected from each municipality using the following criteria: time on the job, time of residence in the neighborhood, and involvement in community activities. In 2016 and 2017 interviews were also conducted with 77 CHWs from eleven primary health clinics in the city of São Paulo. These workers were selected considering differences in age and service time. CHWs were asked to tell stories of families they engage with, describing easy and difficult situations they encountered, how they judge situations, make decisions and justify them. Samples from two time periods revealed stability in CHW practice, continuity in the overarching health policies in which CHW work is embedded, as well as similar socioeconomic vulnerabilities. Fieldwork was approved by the Research Ethics Committee of the Federal University of ABC Region, Brazil.

**Image 1:**
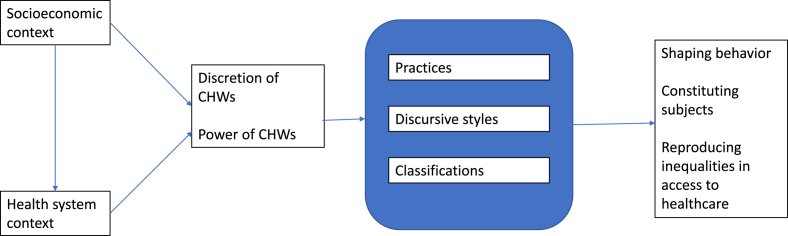
Synthetizes the analytical logic of the paper.

Data was analyzed in NVivo. The first step of coding used a grounded approach and involved identifying the activities carried out by CHWs during implementation (practices), how they interacted with users (discursive styles, based on words and oral expressions used), and how they made judgments about, and categorized, users (classifications). This tripartite analysis uses a rationale similar to Timothy Hoff’s (2013) taxonomy of implementation practices, in which hard practices (measurable and visible procedures; corresponding to our practices) are distinguished from soft practices (relational and discursive mechanisms; corresponding to our discursive styles and classifications). In a second, axial coding, we grouped all the codes according to conceptual categories. (see Image 1)

### 3.3 Practices: what CHWs do

We identified 108 practices – representing what CHWs do when exercising their discretion – and grouped them in 7 categories. Table 1 presents the incidence of each category in relation to the total of practices observed. Overall, 45% of the practices of CHWs are not predicted in any guidelines or regulations – for example, activities related to community mobilization and the claiming of rights. Work is marked by a high degree of discretion and informality, as CHWs constantly grapple with unforeseen demands and constraints in a context of resource scarcity.

**Table 1:** Practices of Brazilian CHWs, as observed during ethnography in Sobral, Taboão da Serra and São Paulo, 2008–2010.

Category of practice	Example	Incidence (%)
Orientation, information and referrals	Explaining the location of services or how to access them	21.6
Team and management support	Participating in management activities in the clinic	20.4
Information management	Filling out forms	15.9
Treatment, prevention and health promotion	Explaining how to prevent diseases	13.6
Mobilization and rights	Engaging users in local council discussions	8.4
Compliance	Checking if patients are taking medicines properly	5.7
Others	Giving information about other services	14.7
Total		100

### 3.4 Discursive styles: how CHWs interact

Discursive styles are relational mechanisms, identified by words and expressions used during interactions. They help to determine the kind of relationship emerging in a specific context (Goffman, 1974; McLean, 2007). They are crucial in the day-to-day activity of CHWs, enabling them to shape how the interaction will unfold – in other words, how health system users are treated and thus how policies are implemented. We identified 24 discursive styles and grouped them in 4 categories. Table 2 shows the percentage of CHWs using each category.

**Table 2:** Discursive styles of Brazilian CHWs, as observed during ethnography in Sobral, Taboão da Serra and São Paulo, 2008–2010.

Category of discursive style	Explanation and examples	% of CHWs
Mediation	Translating information into language that can be understood and remembered Example: in response to a patient who could not read the prescription and did not know how to take the medicines, the CHW mediated the information: ‘this orange-colored pill reminds us of the orange juice we drink in the morning, so you take the orange medicine every morning. And these two white pills you can take with milk, which is also white, when you go to bed’. (CHW 8 Ethn)^a^	77,1
Facilitating understanding	Contextualizing and adapting information to local realities (using references to family, religion or lay knowledge) Example: a CHW tells the patient: ‘what is happening to you is the same that happened to our neighbor, Ana. Do you remember how she was and how I helped her?’. (CHW 23 Ethn)	70
Making relations horizontal	Establishing symmetrical reciprocity and proximity Examples: a CHW tells the patient: ‘you and I we have been through very similar situations in our lives’ or ‘you don't need to thank me. This is only my obligation’ (CHW 12 Ethn)	65
Making relations hierarchical	Establishing distance through authority or magnanimity Example: a CHW tells the patient: ‘I can do this for [your husband], but you have to tell him this is not my obligation’. (CHW 8 Ethn)	38,7

a Citations are identified by the number ascribed to each CHW and specify whether the data was collected during interview (Int) or ethnography (Ethn).

### 3.5 Classifications: how CHWs judge and categorize users

The day-to-day running of health systems requires that its users are classified. Interviews with CHWs included the questions: which types of users do you treat; which users are easier or more difficult to treat; and how do you justify this difference? Answers revealed the coexistence of two processes of judgment and classification. The first type of classification is the official scheme of the health system, which categorizes users according to gender, race, age and type of illness. However, official categories are inadequate when CHWs are forced to make allocation decisions in a context of informality and resource scarcity (something they are not officially mandated to do). CHWs resort to another classification scheme, based on the degree of adherence or resistance to treatments and recommendations. In this scheme, users fall into two categories: ‘easy’ patients, who are committed to treatment, follow recommendations and show respect towards CHWs; and ‘difficult’ ones, who do not follow recommendations, are hostile or do not value CHWs. The former are ‘those who obey us and follow treatment’ (CHW52 Int); ‘those who care about their own health’ (CHW13, CHW38, CHW47 Int); or ‘those who want to be treated’ (CHW9 Int). The latter are referred to as ‘those who do not listen to our advice’ (CHW22, CHW 44 Int); or ‘the irresponsible ones who do not go to the appointments and do not follow treatment’ (CHW19 Int).

Three justifications underpin this unofficial classification. The first, relating to morality and behavior, is visible in statements like: ‘adolescents are irresponsible because they only want to go to the disco and do not take care of their health’ (CHW35 Int); ‘there are mothers who are not careful with their children’ (CHW3 Int); ‘the young pregnant women are irresponsible’ (CHW11, CHW22, CHW45, CHW59 Int). Moral judgements are used to present some patients as docile: ‘the polite elderly’ (CHW7, CHW32, Int); ‘the hardworking people’ (CHW11 Int). The second justification projects a typical socioeconomic and cultural user profile: ‘common and structured families’ (that is, the heteronormative family) (CHW8, CHW18, CHW39, CHW72 Int); ‘the poorest and most humble’ (CHW58 Int). Those from different cultural backgrounds (who are also traditionally poorer), such as immigrants from the Brazilian Northeast, Bolivians and Nigerians, are deemed potentially resistant. Families that do not conform to commonly-observed models – for example single mother households – are deemed potentially problematic. Finally, users are classified as resistant or adherent in line with their pathology, which may help or complicate treatment. Adherent patients are identified with health conditions, like chronic diseases, that make them ‘responsible’ in relation to care and hence ‘deserving’. Resistance is commonly associated with drug users, alcoholics, psychiatric patients, the homeless and people with communication difficulties. The nature of their condition is seen to hinder interaction with CHWs and continuity in treatment.

## 4 The power of CHWs: changing behavior

The analysis of CHW activity shows the multiple sites where power is mobilized and reveals the usefulness of the multilayered notion of domination. Changing behaviour, a typical function of street-level bureaucrats, is most obvious in the day-to-day activity of Brazilian CHWs. Its centrality can be explained by the specific demands and requirements of the ESF, a primary health strategy that seeks to reduce the frequency of hospital-based care by promoting healthy choices and behaviours. Behaviour change is also explained by the Brazilian health system, which combines an aspiration to universality with the realities of diversity, inequality and insufficient resources. This context forces CHWs to establish criteria of prioritization and even inclusion/exclusion for public policies. Changing behaviour often becomes necessary for granting access to benefits and programmes. This happens in the SUS and in relation to other social policies like *Bolsa Família*, the government's cash-transfer scheme with conditionalities related to health behaviours (like the vaccination of children), which CHWs are tasked with monitoring.

The importance of behavior change can be ascertained by the high incidence of practices of ‘Orientation, information and referrals’ (21.6%), ‘Clinical treatment, prevention and health promotion’ (13.6%) and ‘Compliance’ (5.7%). Here, the power of CHWs stems from their unique position at the intersection of three realms: the health system, through which they access expert knowledge and state resources; the community, where they gain awareness of micro-level dynamics and access lay knowledge (including alternative healing practices); and the domestic setting where a significant portion of their practices are carried out. Even though their actual degree of access to people's homes varies (since users are not forced to open their doors), CHWs are almost always able to get an insider knowledge of users' living conditions because they are community members, neighbors, and sometimes even family members. CHWs traverse the private-public divide in ways that other health professionals cannot, and this places them in a privileged position to shape behavior.

CHWs sometimes seek to impose health behavior through appeals to authority and by the distribution of rewards, threats and punishments. This happens when they assume a hierarchical discursive style with non-compliers. 38.7% of CHWs studied seek to shape behavior by establishing hierarchies in their interactions. A CHW was seen threatening a young woman who did not take her pills: ‘I will come back here tomorrow and if you don't take them properly I will tell your parents' (CHW8 Ethn). Another CHW angrily scolded a mother: ‘Why did you have a child if you are not patient enough? If I know you're hitting him, I'll bring the police here’ (CHW12 Ethn). Another told us how she convinced a patient to have her baby vaccinated: ‘I tried to convince her many times [and] she didn't listen to me …. But one day I told her: if you don't have him vaccinated, you will lose the *Bolsa Família*. And then she decided to comply’ (CHW17 Ethn). Hierarchies are also established when CHWs make promises: ‘you take care of your health and I will see if I can get you a job’ (CHW2 Ethn). Even though their in-depth knowledge of users' lives (such as family relations or job situation) is a condition for the success of their interventions, it opens the door to abuses when in attempting to shape behavior CHWs position themselves as powerful in relation to users who are already in situations of vulnerability.

When shaping behavior, CHWs routinely affirm a distinction between ‘me’ or ‘we’ (the health services) and ‘you’ (the users), based on appeals to authoritative knowledge – the idea that ‘we’ know better than ‘you’ about how to live a healthy life. For example, a CHW told a man who did not take his medicines that ‘we do everything we can for you. We know what we are doing because we study to know what to do’ (CHW9 Ethn). In these appeals, behavior is shaped by re-inscribing distance. In other cases, appeals are more ambiguous, based both on science and on CHWs' own experiences and beliefs. Responding to a situation in which a feverish child was being given homemade medicines, the CHW combined his own opinions with official guidelines, advising the mother: ‘homemade medicine is not always the best option. You should give him a spoonful of honey and don't let him walk around without shoes. If that doesn't work, get him to a healer [*rezadeira*] and later bring him to the doctor’ (CHW9 Ethn). Here, to establish proximity with the user, the shaping of behavior mixed appeals to traditional medicine and biomedicine.

## 5 The power of CHWs: producing citizens

The classification of users as resistant or adherent combines the shaping of behavior with the constitution of subjects. It is underpinned by moral judgments and by the expectation of user predispositions to act in certain ways. When classifying users, CHWs do not just seek to change behavior but also advance an ideal subject of care. Good patients are malleable subjects that yield to the influence of CHWs and are predisposed to behave in light of recommendations. Good patients also follow CHW expectations of a desirable moral life. Ideas of motherhood are a good example. One CHW explained how she recognizes a ‘good mother’ (a concept she brought up during the interview): ‘to be a good mother you have to learn how to take care of yourself. We cannot overburden our children. They need to be taken care of’ (CHW27 Int). For another CHW, the most difficult patients are ‘young mothers. They are irresponsible because they do not know who the fathers of their children are and do not have a family structure to care for their children’ (CHW38 Int).

CHWs also advance visions of the ideal beneficiary of the health system. They routinely tell users how they should behave in the clinic, how they should treat CHWs and relate with public authorities. In this way, CHWs help to constitute individuals as subjects of the state. Much of the work of CHWs seeks to enhance the position of users vis à vis the state. Through ‘Mobilization and rights-based activities’, CHWs involve users in community activities and in the life of local institutions. Activities under this rubric may include IT classes for the elderly or support groups for single mothers or victims of abuse. In these, users are recognized as rights-bearing citizens. CHWs help to elucidate how rights can be accessed and how users can participate in policy decisions. A vision of active citizenship, community engagement and belonging is advanced.

In their discursive styles, CHWs determine the degree of proximity or distance between the state and citizens. Discursive styles that hierarchize relations tell users that they know less or are less powerful than state representatives. When CHWs use discursive styles that render relations horizontal, users are brought closer to state authorities. One example is the use of common references, when CHWs mention the names of people or institutions (like churches or associations) known to both interlocutors, thus signaling shared social references. When a CHW declares that ‘every patient is a reflection of his CHW’ (CHW66 Ethn), a particular form of reciprocity is established in which an ideal health system user (and citizen) is projected alongside injunctions towards ‘responsible’ or compliant behaviors. At the same time, the position of the CHW as role model is reaffirmed. As with the shaping of behavior, the task of producing ‘good citizens’ - those that deserve to be taken care of by the state – is done by Brazilian CHWs in a challenging and ambiguous context where aspirations to universal access to public healthcare clash with the reality of insufficient resources. The CHW-as-role model is never separate from the CHW-as-gatekeeper.

## 6 The power of CHWs: reproducing inequality

The activity of Brazilian CHWs is permeated with situations that undermine the health system's vision of universality and equality. For example, in practices of ‘Orientation, information and referrals’, which represent 21.6% of activities, we observed that some CHWs only provide advice ‘by the book’, that is, on services under their responsibility, while others go beyond and offer guidance on informal ways of accessing the system. Some CHWs make personal referrals to other health professionals or ask for favors inside the health clinic for a specific patient. Others seek to solve problems not related to the health system, like helping users find a job or secure a place in school for their children.

The decision between ‘official’ and ‘non-official’ referrals is sometimes connected to practical considerations. Trying to work around constraints or find creative solutions requires effort and time. Given the lack of resources, multiple demands and overwork, it is not always possible to devote this level of attention to users' needs. Many CHWs decide to make their own lives easier and work strictly according to guidelines. In other cases, decisions are based on the degree of deservedness ascribed to users, which in turn is based on classification processes. If users are deemed adherent, CHWs will offer more than the official information and activate other channels, helping citizens access services more easily. For others, the CHW might say: ‘I can't because this is not my obligation’ (CHW29 Int). Since referrals are in part determined by how users are classified by individual CHWs, cases with the same clinical picture can follow different trajectories in the healthcare and public service system. Through their discretion, CHWs have the power to determine who gets what, based on the classifications they attribute to users. Put differently, they can decide who will access resources and who will be excluded.

Classification processes based on moral and behavioral factors, and even on certain health conditions, emerge as potentially exclusionary. Even within the strict remit of CHW responsibility, users are treated differently based on how they are classified. Adherent users, with whom it is possible to build rapport, become deserving and receive more attention and resources (specialist appointments and household visits for example). Resistant users are often considered unworthy of the system's scarce resources. One CHW said: ‘I don't waste appointments with this kind of user’ (CHW17 Int). For some users, vulnerability and exclusion become mutually reinforcing. The high vulnerability of certain groups – such as drug users, pregnant teenagers or patients with psychological disorders – leads to non-compliance with treatments and recommendations. They are thus classified as non-adherent. Nonetheless, their health condition would precisely require more attention. The classification, and its underpinning judgments, exclude a patient profile that should be a priority. In these cases, non-adherence is less an individual choice than further evidence of vulnerability, which is aggravated when CHWs classify these users as undeserving and shut down part of the state's doors to them.

Brazilian CHWs can therefore contribute to the reproduction of existing inequalities in access to health services and in treatment by the health system. This happens in a material sense because CHWs are routinely involved in allocative inclusion and exclusion processes. Even though they are not formally entrusted with gatekeeping roles, CHWs work as part of the ESF, a programme that is designed to make referrals to other levels of care. Moreover, in a context of discretion and resource scarcity CHWs act as *de facto* gatekeepers in the system, making decisions about the eligibility of citizens or imposing sanctions. In practice, their actions and decisions help to determine the quality and quantity of benefits available to users. CHWs also impact upon inequalities in a symbolic way as they judge each situation and classify users, thus deciding who is worthy of receiving services, and what they deserve to receive.

These effects, although stemming from the discretion and power of CHWs, are conditioned by Brazil's deeply contradictory health system. Inequalities in access become the by-product of a system that aspires to universality without sufficient resources; that requires local-level flexibility without providing frontline workers with the training to deal with policy ambiguity and make informed allocation decisions; and that has failed to provide adequate design, implementation and monitorization of policies to respond to great inequality and heterogeneity. In this situation, the role of the CHW is ambiguous, oscillating between the goal of expanding access and the reality of demand management. On the one hand, CHWs are deployed to open the doors of the system to the most vulnerable groups; on the other, they end up functioning as the system's foremost gatekeepers.

By reproducing inequalities in access to health, and by treating users differently, CHWs can contribute to reinforcing broader social vulnerabilities. The Brazilian CHW programme has been accused of contributing to a chasm between technologically-developed services for privileged groups and a ‘low-tech’ (and frequently under-resourced) assistance to the poor (Favoreto and Camargo, 2002). According to this interpretation, and while they provide essential services to groups who would otherwise be neglected, CHWs end up becoming a ‘band-aid’ that epitomizes the structural exclusion of the poor from the highest standards of healthcare. Whilst everyone can benefit from CHW visits, this does not necessarily mean access to the full range of services available in the public health system – for example, specialist appointments have been identified as a ‘bottleneck’ (Spedo et al., 2010). The number of appointments that CHWs can provide is well below actual needs, and poor people can wait years to access services that others will pay for in the private sector. Brazilian CHWs are co-opted in the reproduction of a system that, in its current form, fails to respond to deep-seated vulnerabilities and breeds socioeconomic inequalities.

## 7 Conclusion

This article analyzed the role of power in policy implementation. Combining street-level bureaucracy with a notion of power as domination, we showed that implementers are not powerless or apolitical, neither work simply as diplomats persuading or engaging actors (Gale et al., 2017). Rather, they exercise power in asymmetric relations. These findings are relevant to the analysis of implementation in situations of informality, where proximity between implementers and users, and the discretion of the former, is encouraged as a pathway for ‘context-specific’ and ‘culturally-sensitive’ interventions. Informality has the potential to lead to unequal power relations, particularly when combined with inadequate training and resources.

The article focused on Brazil's community health workers. We explored what these workers do, how they interact with users, how they make decisions and how these decisions are justified. We argued that their work is marked by discretion in a context of informality and resource scarcity, thus providing ample space for the exercise of power. The power of CHWs operates not simply by shaping behavior. It also constitutes subjects and reproduces inequality in terms of how users are received by the health system, and their degree of access to the range of services available. The power of Brazilian CHWs is visible not only in decisions about which practices they will carry out, how and towards whom, but also in constituting individuals as ‘deserving’ or ‘underserving’. CHWs use unofficial classification schemes based on personal worldviews, their expectations about the profession, their perceptions of how well they are valued by users, their moral conceptions of what is right or wrong, and stereotypes about the poor and vulnerable (even though many CHWs experience the same vulnerabilities). These classifications help to determine access to the health system and shape how users are treated if access is granted. These findings question an overwhelmingly positive view of the impact of CHWs in terms of promoting social justice and empowerment (Becker et al., 2004; Pérez and Martinez, 2008; Ingram et al., 2008). We demonstrated that the picture is more complicated, and that the concrete workings of power relations need to be scrutinized when assessing the impact of CHW programmes.

The case of Brazil shows that the (unregulated) mobilization of power by implementers can be, at least in part, complicit in the reproduction of inequality in access to, and treatment by, the health system. In this sense, inequality is reproduced not because of the failure or absence of policy, but as a by-product or unintended (and sometimes unnoticed) consequence of the policies designed to alleviate inequality. CHW discretion and power cannot be separated from the Brazilian context of resource scarcity, economic inequalities, heterogeneity and extremely high levels of demand. This context places tremendous pressures upon CHWs. While being asked to alleviate inequality by expanding access and reaching out to vulnerable groups, CHWs end up functioning as *de facto* gatekeepers to healthcare and public services, making crucial decisions about who gets what – despite not being trained or mandated to do so.

The wide scope for CHW discretion and power reflects the tensions, ambiguities and contradictions of the Brazilian health system. It is partly the result of insufficient resources and deficient coordination, but also a way to enable responsiveness in the face of heterogeneity. CHW discretion can provide the health system with greater flexibility and resilience, helping it adapt to multiple and changing demands. In Brazil, CHWs can lead to more inclusive policies when they engage in non-hierarchical ways with different user experiences and forms of knowledge, and when they mediate and translate policies to facilitate understanding by users. CHWs also promote inclusivity by allowing the system to take on board the heterogeneity of territories and users.

On the other hand, the discretion of Brazilian CHWs has led to adverse effects. These can be partly explained by inadequate training of CHWs, who are unprepared to make allocation decisions. Lack of attention and control over CHW discretion, and insufficient awareness of the power mobilized by CHWs, also reinforces the exclusionary and inequality-inducing side-effects of policies. The discretion and power of CHWs should be closely considered during the design, management and evaluation of policies, as well as in the training of all health professionals. An awareness of the potential pitfalls of CHW activity is a crucial step towards designing and implementing health policies that can effectively reduce inequality.
